# Taxonomic note on *Trichelix horrida* (Pfeiffer, 1863) from Laos, with a type catalogue of *Moellendorffia*, *Trichelix*, and *Moellendorffiella* (Heterobranchia, Camaenidae)

**DOI:** 10.3897/zookeys.952.52695

**Published:** 2020-07-23

**Authors:** Chirasak Sutcharit, Khamla Inkhavilay, Somsak Panha

**Affiliations:** 1 Animal Systematic Research Unit, Department of Biology, Faculty of Science, Chulalongkorn University, Bangkok 10330, Thailand Chulalongkorn University Bangkok Thailand; 2 Department of Biology, Faculty of Natural Science, National University of Laos, P.O. Box 7322, Dongdok, Vientiane, Laos National University of Laos Vientiane Lao People's Democratic Republic

**Keywords:** Biodiversity, Indochina, Japan, land snail, limestone, systematics, type specimen

## Abstract

Land snail surveys conducted in northern Laos between 2013 and 2014 have led to the discovery of a living population of *Trichelix horrida* (Pfeiffer, 1863). This species has never been recorded from specimens other than the types, and its distribution and anatomy have remained essentially unknown. The genitalia and radula morphology are documented here for the first time and employed to re-assess the systematic position of this species: the unique morphological characters of *T. horrida* are a penis similar in length to the vagina, a small and triangular penial verge, gametolytic organs extending as far as the albumen gland, head wart present, and unicuspid triangular radula teeth. The type locality of this species was believed to be from “Lao Mountains, Camboja,” and is restricted herein to be Luang Phrabang Province, northern Laos. The assignment of species to either of three genera, *Trichelix* Ancey, 1887, *Moellendorffia* Ancey, 1887, and *Moellendorffiella* Pilsbry, 1905, based solely on information provided in their original descriptions is difficult. The type specimens of all nominal species presently placed in either of these three genera are examined and illustrated herein. Comparison with the primary type specimens will assist future revisions aiming to resolve the systematics of these taxa. In addition, we transfer *Moellendorffia faberiana* (Möllendorff, 1888) to the genus *Moellendorffiella*.

## Introduction

The land snail genus *Trichelix* Ancey, 1887 has a wide distribution from southeastern China to the northern parts of Laos and Vietnam, Taiwan, and the central Ryukyu Islands of Japan (Schileyko 2003). Originally, *Trichelix* was described as a monotypic genus for the type species *Helix horrida* Pfeiffer, 1863. The flattened to sunken spire, elevated parietal callus, aperture with denticles, external furrows on the outer wall of the last whorl, and the hirsute shell microsculpture confer a very distinctive morphology to the shell of this species (Ancey 1887; Pilsbry 1890, 1895, 1901, 1905; Yen 1939; Zilch 1960). The early taxonomic work was restricted to the description of shell morphology, and Pilsbry (1905) treated *Trichelix* as a subgenus of *Moellendorffia* Ancey, 1887 due to a similar shell and apertural morphology. Five additional species have since been assigned to this taxon (Pilsbry 1905). Subsequently, Habe (1957), Minato (1971, 1980, 2011), and Azuma (1982) described the genital anatomy of the species from the eastern Asian islands. Schileyko (2003) revised the genus based on published information about species other than the type species. Schileyko (2003) found differences in the genital anatomy of species, which may be indicative of a distinct lineages, but he hesitated to propose this conclusion because the anatomy of the type species was still unknown. Later, examination of the genital anatomy and shell morphology of *Moellendorffia eastlakeana* (Möllendorff, 1882) from Vietnam has suggested a possibly close relationship between *Moellendorffia* and *Trichelix* (Panha et al. 2010). Recently, Minato (2011) reviewed the genus and followed Pilsbry’s (1905) classification by recognizing *Trichelix* as a subgenus; Minato (2011) examined the genital anatomy of Taiwanese species. These reports appear to be the only published literature on the systematics of *Trichelix*.

*Helix horrida* Pfeiffer, 1863 was established based on three specimens from the collection of H. Cuming, and these syntypes were collected by H. Mouhot. The type locality was stated to be “Lao Mountains, Camboja,” without any other precise locality information. Localities recorded by Mouhot were usually tentative and based on a broad geographical scale. This has rendered it difficult to infer the type localities of many species that were described based on material collected by Mouhot, including fish (Kottelat and Tan 2018), reptiles and amphibians (Stuart et al. 2006), and land snails (Sutcharit et al. 2019). Similarly, the type locality of *Helix horrida* Pfeiffer, 1863 is rather vague, and with no later records of this species available. The distribution of this species has remained essentially unknown to this day. The field surveys performed during 2013 and 2014 in the northern part of Laos contained a records of *Helix horrida* Pfeiffer, 1863 and comparisons with the type material confirm its identity.

Here, we report on the examination of examples of *T. horrida* (Pfeiffer, 1863) collected from northern Laos. The type locality is discussed, and a correction is proposed in accordance with the guidelines of ICZN (1999). In addition, the primary type specimens of all recognized taxa belonging to the genera *Moellendorffia*, *Trichelix*, and *Moellendorffiella* Pilsbry, 1905 are included for comparisons and because the species identification could not have been possible without comparison with the type specimens.

## Materials and methods

Shells and living specimens were collected in a limestone forest in Luang Phrabang Province, northern Laos. The live specimens were photographed, euthanized (AVMA 2013), and then transferred to 70% (v/v) ethanol for fixation and preservation. The genitalia of three specimens were dissected and examined under a stereomicroscope. Drawings were made with a camera lucida. Radulae were extracted, soaked in 10% (w/v) NaOH, and then examined under scanning electron microscopy (SEM; JEOL, JSM-6610 LV). The formulae and morphology of radula were observed, recorded, and described. Adult shells were used to measure the shell height and shell width, and to count the number of whorls. The voucher specimens are now deposited in the Chulalongkorn University Museum of Zoology (CUMZ) and in the collection at the National University of Laos.

Anatomical conventions and abbreviations: In the descriptions of the genitalia, the following abbreviations are used, as defined by Habe (1957), Schileyko (2003), and Panha et al. (2010). The term ‘proximal’ refers to the region closest to the genital orifice, while ‘distal’ refers to the region furthest away from the genital orifice. Abbreviations: ag, albumen gland; at, atrium; e, epiphallus; fl, flagellum; fo, free oviduct; gd, gametolytic duct; gs, gametolytic sac; hd, hermaphroditic duct; hw, head wart; ov, oviduct; p, penis; pp, penial pilaster; pr, penial retractor muscle; pv, penial verge; v, vagina; vd, vas deferens; vp, vaginal pilaster.

### Institutional abbreviations

**ANSP** Academy of Natural Sciences of Drexel University, Philadelphia

**MCZ** Museum of Comparative Zoology, Harvard University, Cambridge

**MNHN** Muséum National ďHistoire Naturelle, Paris

**NHM** Natural History Museum, London

**NHMW** Naturhistorisches Museum, Vienna

**NIGPAS** Nanjing Institute of Geology and Paleontology, Chinese Academy of Sciences, Nanjing

**SMF** Forschungsinstitut und Naturmuseum Senckenberg, Frankfurt am Main

**ZMB** Museum für Naturkunde, Berlin

**ZMNH AIMS** Zhejiang Museum of Natural History, Hangzhou

### Photo credits

Photos of the type specimens from the Molluscs Collection (IM) of MNHN are credited to the museum taken under the project E-RECOLNAT: ANR-11-INBS-0004 and MNHN/Philippe Maestrati, or as otherwise stated.

## Systematics

### Family Camaenidae

#### Genus Trichelix

Taxon classificationAnimaliaStylommatophoraCamaenidae

Ancey, 1887

693D028C-C37E-57D9-8967-90CBCA0F24F7

Trichelix Ancey 1887: 64. Schileyko 2003: 1513.
Helix (Trihelix) : Pilsbry 1890: 9 (incorrect subsequent spelling). Pilsbry 1895: 289.
Moellendorffia (Trihelix [sic]): Pilsbry 1905: 65.
Moellendorffia (Trichelix) : Zilch 1960: 612.

##### Type species.

*Helix horrida* Pfeiffer, 1863; by original designation.

##### Description.

Shell small to medium-sized, flattened to concave, rather thin, umbilicate, and corneous to brownish. Spire shrunken; embryonic shell nearly smooth; following whorls granulated and with short to long periostracal hairs arranged in oblique rows along the lines of growth. Last whorl rounded and descending anteriorly. Aperture ventral or subvertical; trigonal or subcircular; with strong or weak barriers inside the aperture at upper periphery and below periphery, and externally marked with strong to weak longitudinal furrows. Peristome expanded and continuous or discontinuous; parietal callus thin or thickened and little elevated.

Genitalia typical of camaenids, without either dart apparatus or accessory glands. Penis and epiphallus long, penial verge present, and flagellum short. Internal wall of penis and vagina with longitudinal pilasters.

Radular teeth arranged in V-shaped rows; central and lateral teeth triangular.

##### Remarks.

The genus is currently comprised of six nominal species (Schileyko 2003; Minato 2011). Two species occur in northern Laos and southern China (Fig. 3), viz. *T. horrida* and *T. biscalpta* (Heude, 1885), and one has been recorded from Taiwan, viz. *T. hiraseana* (Pilsbry, 1905). Three species occur on the Amami Islands, Central Ryukyu Islands, Japan, viz. *T. eucharista* (Pilsbry, 1901), *T. diminuta* (Pilsbry & Hirase, 1905) and *T. tokunoensis* (Pilsbry & Hirase, 1905).

#### Trichelix horrida

Taxon classificationAnimaliaStylommatophoraCamaenidae

(Pfeiffer, 1863)

1022CAD3-022A-5EDA-841B-929249734061

Figures 1 , 2 , 7B

Helix horrida Pfeiffer 1863[“1862”]: 272, pl. 36, fig. 15. Type locality: “Lao Mountains, Camboja” [probably in northern Laos around Luang Phrabang area, Laos]. Pfeiffer 1868a: 395. Pfeiffer 1868b: 399, 400, pl. 92, figs 17–19. Pfeiffer and Kobelt 1880: 579, pl. 170, figs 8–10.
Helix (Trihelix) horrida : Ancey 1887: 64. Pilsbry 1890: 9, pl. 1, figs 9–11.
Helix (Moellendorffia) horrida : Pilsbry 1895: 290.
Moellendorffia (Trichelix) horrida : Zilch 1960: 612. Inkhavilay et al. 2019: 105, figs 53f, 54a, 58h.
Moellendorffia horrida : Richardson 1985: 185.

##### Type material.

Three specimens originating from H. Cuming’s collection with the original label stating the taxon name and collection location in Pfeiffer’s handwriting are present in the malacological collection of the NHMUK. Of these specimens, the one most closely matching the measurements given in the original description is here designated as the lectotype NHMUK 20200202/1 (Fig. 7B) to stabilize the name. The other two shells from the same lot become paralectotypes NHMUK 20200202/2 to 20200202/3.

*Trichelix horrida* was originally described based on specimens collected by H. Mouhot, with “Lao Mountain, Camboja” as the published type locality. Our survey following Mouhot’s itinerary in the south-western part of Cambodia yielded no specimens that could be identified in this genus. This record type locality seems to be imprecise. On the other hand, our survey in the northern part of Laos, where Mouhot had visited Luang Phrabang in 1861, recorded populations of this species in Muang Ngoi about 90 km north of Luang Phrabang City. Therefore, we restricted the known distribution and propose Luang Phrabang Province, Laos as the correct type locality for this species.

##### Material examined.

**Figure 1.**
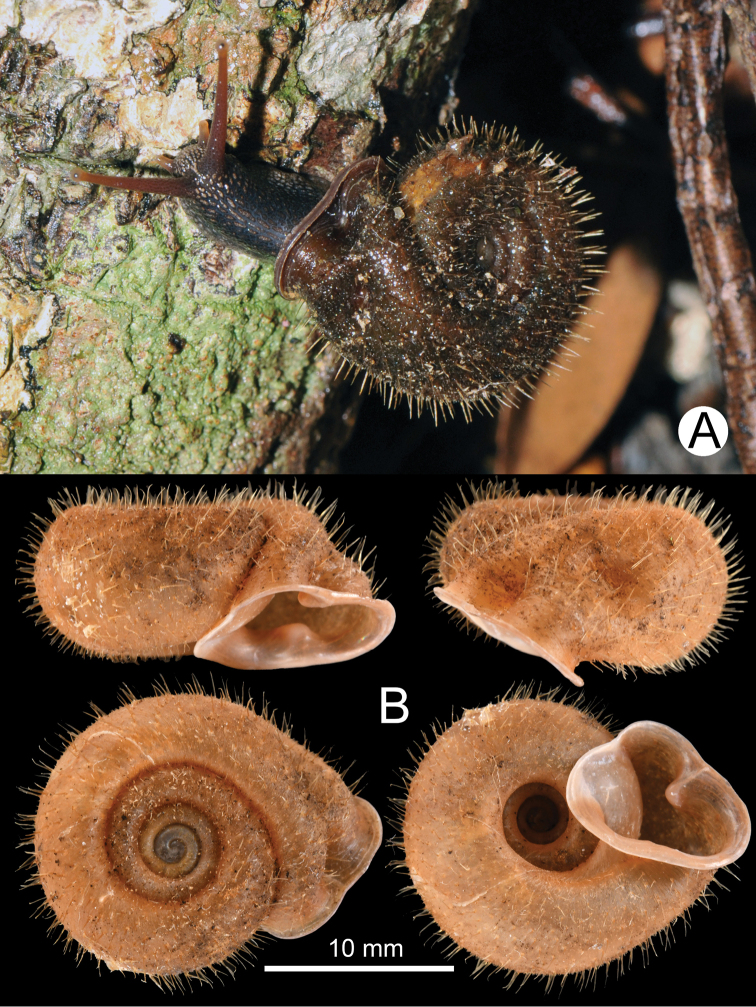
Living snail and shell **A** specimen CUMZ 5248 **B** specimen CUMZ 5249.

Moist evergreen forest on limestone hills between Ban Pha Toke and Ban Nong Ian, Muang Ngoi (Town), Ngoi District, Luang Phrabang Province, Laos (20°32'31.2"N, 102°38'56.3"E): CUMZ 5248 (eight specimens in ethanol; Fig. 1A), CUMZ 5249 (five shells; Fig. 1B), CUMZ 5250 (one shell).

##### Measurement.

From 10 specimens analyzed; shell height ranged from 12.4–14.7 mm (mean 13.5 ± 1.0); shell width ranged from 20.8–23.9 mm (mean 22.0 ± 1.2); and whorl count ranged from 6–6⅛ whorls.

##### Shell.

Shell medium-sized, dextral, slightly thin, translucent, depressed globose, biconcave shaped (dorsoventrally concave), and deeply umbilicate. Whorls 5–6, slightly convex, and increasing regularly; suture depressed, spire concave, looking like umbilicus. Embryonic shell large with very fine growth lines. Following whorl with corneous to brownish periostracum; upper surface with long hairs arranged in oblique rows; lower surface with slightly shorter hairs and few hairs around umbilicus. In worn specimens, shell surface possesses rough rows of tubercles running obliquely and descending, relatively smooth around umbilicus. Last whorl well rounded and little convex below periphery. Last whorl descending about ¼ whorl from aperture, and constriction occurs close to apertural lip. Aperture ear-shaped and opened subventrally; lip margin pale corneous, little thickened, and continuously expanded. External furrow aligns with internal apertural lamella or fold. Upper periphery marked with two furrows arranged spirally and correspond with palatal lamella and fold; below periphery with one furrow close to lip aligned with basal lamella. Parietal callus thickened, elevated, emarginated, and obtusely projecting inward. Umbilicus wide, but narrower than apex side which is cascade-shouldered.

##### Genitalia.

**Figure 2.**
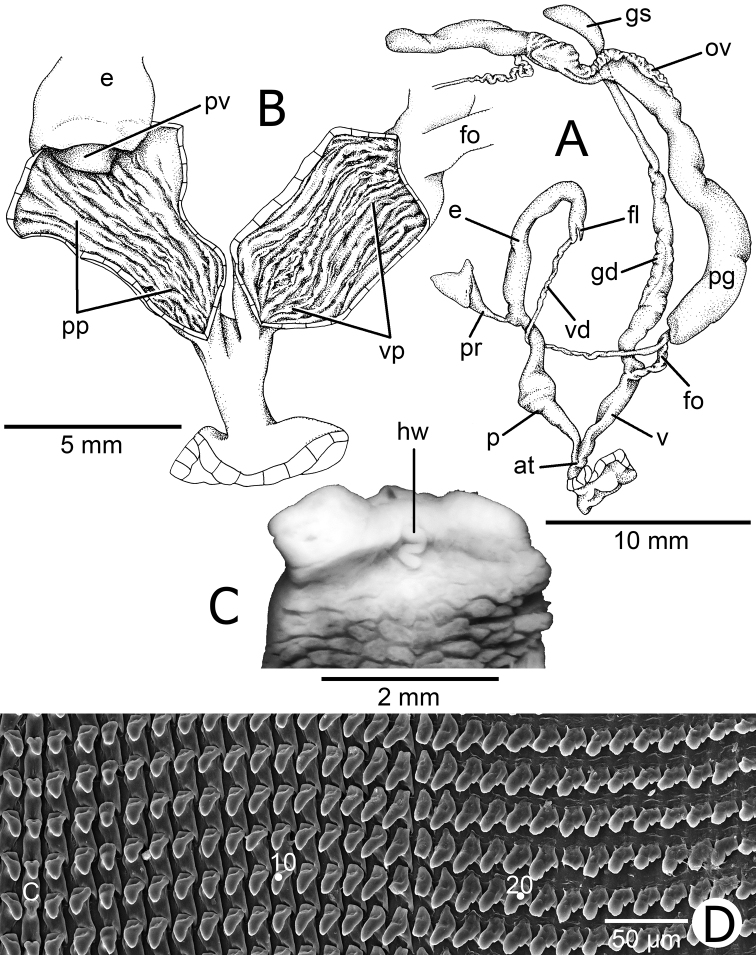
Genitalia and radula of *T. horrida* specimen CUMZ 5248 **A** General view of the genital system **B** Interior structure of the penis and vaginal chambers **C** Dorsal view showing head wart **D** Scanning electron micrographs of central, lateral and marginal teeth. Central tooth is indicated by ‘C’ and the other numbers indicate the order of lateral and marginal teeth.

Atrium (at) short; penis (p) long; proximally with penial verge and enlarged fold at penial verge base; distally similar in length as proximally and somewhat slender tube. Epiphallus (e) slightly enlarged and almost the same length as penis. Flagellum (fl) very short and small. Vas deferens (vd) a small tube, follows vagina and penis, and connects distally on epiphallus and free oviduct. Penial retractor muscle (pr) slightly thickened and long (Fig. 2A).

Internal wall of penis ribbed by a series of swollen longitudinal penial pilasters (pp). Smooth pilasters line introverts penial chamber and encircles penial verge tip. Penial verge (pv) small, short conic with smooth surface (Fig. 2B).

Vagina (v) of similar length to proximal penis and held in position by series of muscles attached to foot floor. Gametolytic organ (duct and sac) long, cylindrical, and extending as far as albumen gland. Gametolytic duct (gd) as wide as gametolytic sac (gs) for most of its length but narrows before reaching gametolytic sac. Free oviduct (fo) short, about half of vagina length; oviduct (ov) small. Prostate gland (pg) and oviduct (ov) developed; hermaphroditic duct long and convoluted tube; albumen gland solid and tongue shape (Fig. 2A).

Internal wall of vagina possesses several longitudinal vaginal pilasters (vp). Pilasters with smooth surface and line entire vaginal chamber (Fig. 2B).

##### Animal.

Live animal covered with blackish-brown reticulated skin and dorsally with whitish stripe in middle of the body. A small curved head wart (hw) is located between the posterior tentacles (Fig. 2C). Foot narrow and long; mantle edge greyish; tentacles brownish, and lower tentacles pale brown. Mantle cavity possesses blackish pigmentation. Live snails possess short to long periostracal hairs, which mostly disappear in worn shells or old snails.

##### Radula.

Teeth arranged in anteriorly pointed, V-shaped rows; each row contains about 75 (37-(18–20)-1-(18–20)-38) teeth. Central tooth unicuspid, triangular with blunt cusp. Lateral teeth unicuspid, triangular with blunt tip, gradually taller laterally and little inclined to central tooth. Marginal teeth starting around tooth numbers 18 to 20 outwards from lateral teeth. Tricuspid or bicuspid marginal teeth, endocone usually absent; mesocone large, broad and with curved to blunt cusp; ectocone slightly large, pointed head and located at base of the teeth. Outer marginal teeth rather small; mesocone and ectocone indistinguishable, with undulated cusp (Fig. 2D).

##### Distribution.

*Trichelix horrida* was previously known only from the type locality (“Lao Mountain, Cambojia” [Cambodia or Laos]). The specimens examined herein were collected from limestone karst in Muang Ngoi Town, about 90 km north of Luang Phrabang City.

Our sampling locality was characterized by monsoonal karst landforms with high humidity. The snails occurred in tropical moist deciduous forest. There was heavy rain before our visit in August 2014. The snails were active, crawling or sitting on moist rotten logs among the limestone outcrops.

##### Remarks.

*Trichelix horrida* is distinctly different in shell morphology from all other *Moellendorffia* species by having a concave spire, rounded last whorl, and two furrows arranged spirally on the upper periphery (Table 1). In contrast, *Moellendorffia* species tend to have flattened to elevated spires, rounded to shouldered last whorls, and two furrows arranged vertically on the periphery. *Trichelix horrida* differs from the other congeners in having two short furrows on the last whorl and an elevated parietal callus (Fig. 7B), while *T. biscalpta* and *T. hiraseana* tend to have a long furrow on the last whorl and unelevated parietal callus (Figs 6C, 7A). In addition, *T. hiraseana* has a relatively long, drumstick-shaped flagellum, while the type species has a very short protrusion (see Minato 2011 for a comparison). In addition, *Chloritis bifoveata* (Benson, 1850) from Myanmar and Thailand, *C. diplochone* Möllendorff, 1898 from Laos and Thailand, and *C. vinhensis* Thach & Huber, 2018 from Vietnam differ from *T. horrida* by having a thin parietal callus, with a shell constriction occurring about half a whorl from the aperture (absent in *C. vinhensis*), and without apertural dentition (Sutcharit and Panha 2010; Páll-Gergely and Neubert 2019; Páll-Gergely et al. 2020).

#### 

**Table 1.** Comparison of *Trichelix* (continental and eastern Asian islands species) and the related genera *Moellendorffia* and *Moellendorffiella*.

Characters	*Moellendorffia* Ancey, 1887	*Trichelix* Ancey, 1887		*Moellendorffiella* Pilsbry, 1905
		Continental group	Central Ryukyu group	
Shell shape	low conic to convex	concave	concave	flat
Last whorl	round or angular	round	round	shouldered or strong shouldered keel
Periostracal hair	short to long	short to long	short	absent
Furrow on upper periphery and alignment on last whorl	–	one or two / spiral alignment	–	–
Furrow on periphery and alignment on last whorl	two / vertical alignment	–	–	one/ spiral alignment
Furrow below periphery	one and strong	one and strong	absent or very weak	one and strong
Parietal callus	long elevated with nodule	short elevated with nodule	thin with cords	thin
Distribution (Fig. 3)	Southern China and Indochina	Central China, Indochina and Taiwan	Restricted to the Central Ryukyu Islands, Japan	Central China
Suggested nominal species (**bold** = type species)	*blaisei*, *deflexa*, *dengi*, *depressispira*, *eastlakeana*, *hensaniensis*, *loxotata*, *messageri*, *sculpticoncha*, *spurca*, ***trisinuata***	*biscalpta*, *hiraseana*, ***horrida***	*diminuta*, *eucharista*, *tokunoensis*	***erdmanni***, *faberiana*

## Discussion

**Figure 3.**
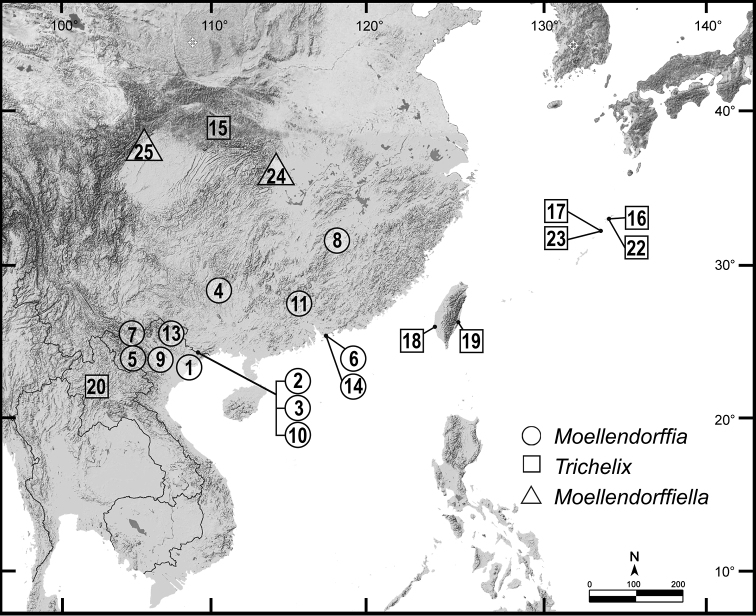
Approximate geographical position of the type locality of all nominal species of the genera *Moellendorffia* (circle), *Trichelix* (square), and *Moellendorffiella* (triangle). The numbers indicated correspond to the nominal species listed in the catalogue of the type specimen.

The newly collected material from Laos presents valuable additional information for the taxonomic position of *Trichelix* and its congeners. The relationship of *Trichelix* with *Moellendorffia* and *Moellendorffiella* has been suggested based on shell and genital anatomy characters (Panha et al. 2010). The shrunken spire and one or two furrows located on the upper periphery are the unique characteristics of *Trichelix*. At present, *Trichelix* s.l. has a wide distribution across Indochina to Taiwan, southern China, and the Central Ryukyu Islands of Japan (Fig. 3).

The genus *Trichelix* s.l. appears to be a heteromorphic assemblage, as noted by Schileyko (2003), based on both shell and genital anatomy characters. The genus comprises the continental group and the Central Ryukyu group. The continental group includes three nominal species from northern Laos (the type species), Taiwan (*T. hiraseana*), and southern China (*T. biscalpta*). They all have prominent palatal lamellae arranged spirally on upper periphery, strong columellar lamella, and vagina almost the same length as penis. The Central Ryukyu group contains three nominal species: *T. eucharista*, *T. tokunoensis*, and *T. diminuta*; they lack the parietal lamella and have a very weak or absent columellar lamella, and the vagina is relatively longer than the penis (Habe 1957; Minato 1971, 1980, 2011; Schileyko 2003). The unique genital characters of *T. eucharista* are: penis about half of vagina length and vagina with constrictions; *T. tokunoensis* possesses two penial retractor muscles, a very small epiphallus and penis about one-third of the vagina length; *T. diminuta* has the penis about half of the vagina length and the gametolytic duct bears constrictions (Habe 1957; Minato 1971, 1980, 2011; Schileyko 2003). These unique and distinct genital characters are likely to be apomorphic traits and would be the main reproductive barrier among these species. It is very likely that the three species inhabiting the Central Ryukyu Islands of Japan do not belong to the same genus as the continental and Taiwanese species. However, with so few synapomorphic traits among these Central Ryukyu Islands species, the confidence in defining distinct lineages remains low. Therefore, we refrain from describing a genus without additional evidence from molecular analyses.

## Catalogue of type specimens of *Moellendorffia*, *Trichelix*, and *Moellendorffiella*

In the following catalogue list, the primary type specimens (i.e., holotype, lectotype, and syntype/s) along with secondary type specimens (paratype/s and paralectotype/s) of *Moellendorffia*, *Trichelix*, and *Moellendorffiella* species are provided. The species-group names are arranged by alphabetical order. The references for the usage of each taxon name have been comprehensively provided by Richardson (1985), Zilch (1966), and Minato (2011). The name in the original combination is given with the bibliographic information or the original description. The type locality is given, and if possible, the modern name and/or regional names of the type locality are provided in square brackets. The current taxonomic status includes the generic placement, whether a valid name or synonym. If necessary, remarks are given on the status of type specimens, authorships, availability of name, notes on the type locality, and other useful comments.

### Alphabetical list of the taxa

**I. Genus *Moellendorffia* Ancey, 1887**

*Moellendorffia*Ancey 1887: 64. Zilch 1960: 611, 612. Schileyko 2003: 1514, 1515.

*Proctostoma*Mabille 1887b: 102.

Moellendorffia (Moellendorffia): Pilsbry 1905: 65. Zilch 1960: 612. Zilch 1966: 210.

**Type species.**

*Helix trisinuata* Martens, 1867; by original designation.

**Diagnosis.**

Shell flattened to globose-conic, and umbilicate. Periostracum thick and covered with short to long hairs. Last whorl rounded to shoulder and descending anteriorly. Aperture trigonal or squarish, entirely free from preceding whorl; with barriers inside, and externally marked with furrows. Parietal wall elevated to form prominent nodule; one or two palatal lamellae (two lamellae arranged vertically); one columellar lamella.

**Remarks.**

The genus *Moellendorffia* can be distinguished from *Trichelix* s.l. in having low conical to elevated spire, one or two furrows (arranged vertically) on periphery and elevated parietal callus, while *Trichelix* s.l. has a concave spire. In addition, the continental-*Trichelix* have one or two furrows (arranged spirally) on the upper periphery and little elevated parietal callus, and the Central Ryukyu-*Trichelix* performs very weak or absent furrows, and a thin parietal callus.

**1. *blaisei* Dautzenberg & Fischer, 1905**

*Moellendorffia blaisei*Dautzenberg and Fischer 1905: 99, 100, pl. 3, figs 17–19. Type locality: Ile Krieu, Tonkin [Krieu Island, Ha Long Provincial, Quang Ninh Province, Vietnam]. Schileyko 2011: 43.

**Current taxonomic status.**

*Moellendorffia*. Valid species.

**Type specimens.**

**Figure 4.**
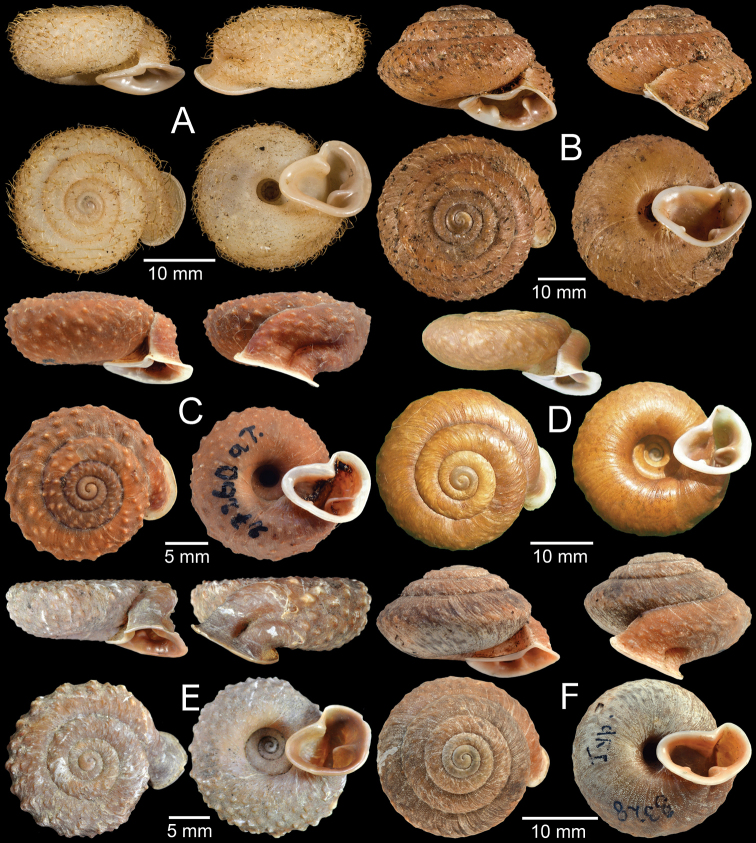
**A***Moellendorffia blaisei*, syntype MNHN-IM-2000-1843 **B***Moellendorffia callitricha*, syntype MNHN-IM-2000-2006 **C***Moellendorffia spurca deflexa*, lectotype SMF 27260a **D***Moellendorffia dengi*, holotype ZMHN AIMS 1693 **E***Moellendorffia depressispira*, syntype MNHN-IM-2000-34941 **F***Moellendorffia eastlakeana*, lectotype SMF 8328/1. Photo: J He (**D**).

Syntype MNHN-IM-2000-1843 (one shell, Fig. 4A).

**2. *callitricha* (Bavay & Dautzenberg, 1899)**

Helix (Moellendorffia) callitrichaBavay and Dautzenberg 1899: 35, 36, pl. 1, fig. 6. Type locality: That-Khé [That Khe Town, Trang Dinh District, Lang Son Province, Vietnam].

*Moellendorffia callitricha*: Richardson 1985: 183.

**Current taxonomic status.**

*Moellendorffia*. Synonym of *Moellendorffia eastlakeana* (see Panha et al. 2010).

**Type specimens.**

Syntype MNHN-IM-2000-2006 (one shell, Fig. 4B).

**3. *deflexa* Möllendorff, 1901**

*Moellendorffia spurca deflexa*Möllendorff 1901: 74. Type locality: Masongebirge [Mau Son Mountains, Lang Son Province, Vietnam].

Moellendorffia (Moellendorffia) spurca deflexa: Zilch 1966: 210, pl. 6, fig. 54.

*Moellendorffia spurca deflexa*: Richardson 1985: 186. Schileyko 2011: 44.

**Current taxonomic status.**

*Moellendorffia spurca*. Accepted subspecies.

**Type specimens.**

Lectotype SMF 27260a (Fig. 4C) and paralectotype SMF 27260b (one shell) from Manson Gebirge, Tonkin.

**Remarks.**

The lectotype was designated in Zilch (1966: 210).

**4. *dengi* Yang, Fan, Qiao & He, 2012**

*Moellendorffia dengi*Yang et al. 2012: 32, fig. 1. Type locality: Leye Country, Guangxi Province, China.

**Current taxonomic status.**

*Moellendorffia*. Valid species.

**Type specimens.**

Holotype ZMHN AIMS 1693 (Fig. 4D) and paratypes unnumbered (three shells).

**5. *depressispira* (Bavay & Dautzenberg, 1909)**

Helix (Moellendorffia) depressispiraBavay and Dautzenberg 1909b: 244. Type locality: Pac-Kha [Pa Kha in Long Luong Commune, Van Ho District, Son La Province, Vietnam]. Bavay and Dautzenberg 1909a: 197, 198, pl. 8, figs 10–12.

*Moellendorffia depressispira*: Richardson 1985: 183. Schileyko 2011: 44.

**Current taxonomic status.**

*Moellendorffia*. Valid species.

**Type specimens.**

Syntype MNHN-IM-2000-34941 (one shell, Fig. 4E).

**6. *eastlakeana* (Möllendorff, 1882)**

*Helix eastlakeana*Möllendorff 1882: 185. Type locality: Guang-dung [Guangdong, China].

Moellendorffia (Moellendorffia) eastlakeana: Zilch 1966: 210, pl. 6, fig. 52.

*Moellendorffia eastlakeana*: Richardson 1985: 184. Panha et al. 2010: 21–24, figs 1–10. Schileyko 2011: 44.

**Current taxonomic status.**

*Moellendorffia*. Valid species.

**Type specimens.**

Lectotype SMF 8328/1 (Fig. 4F) and paralectotype SMF 8329 (one juvenile) from Tai-mo-Shan, Guong-dong.

**Remarks.**

The lectotype was designated in Zilch (1966: 210).

**7. *exasperata* (Bavay & Dautzenberg, 1909)**

Helix (Moellendorffia) loxotata var. exasperataBavay and Dautzenberg 1909a: 196, pl. 8, figs 13, 14. Type locality: Nat-Son, Muong-Hum [probably in the area of Lao Cai Province, Vietnam].

*Moellendorffia loxotata exasperata*: Schileyko 2011: 44.

**Current taxonomic status.**

*Moellendorffia loxotata*. Accepted subspecies.

**Type specimens.**

**Figure 5.**
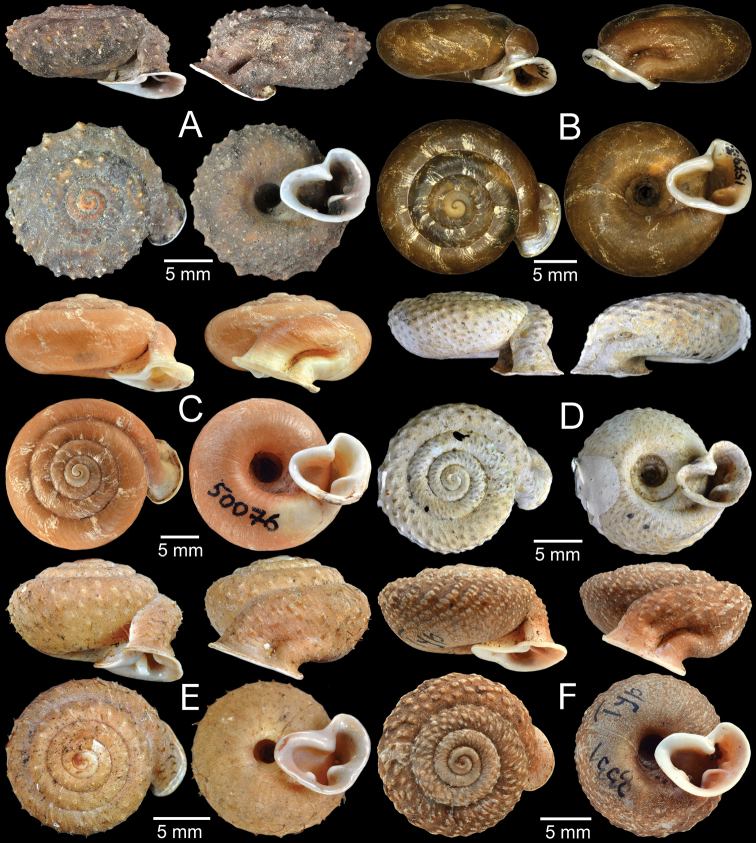
**A***Moellendorffia loxotata exasperata*, syntype MNHN-IM-2000-34940 **B, C***Moellendorffia hensaniensis***B** lectotype NHMW 15795 and **C** paralectotype SMF 50076/1 **D***Moellendorffia loxotata*, syntype MNHN-IM-2000-2071 **E***Moellendorffia messageri*, syntype MNHN-IM-2000-1939 **F***Moellendorffia trisinuata sculpticoncha*, lectotype SMF 8331/1. Photo: S Schnedl (**B**).

Syntype MNHN-IM-2000-34940 (one shell, Fig. 5A).

**8. *hensaniensis* (Gredler, 1885)**

Helix (Polygyra) hensaniensisGredler 1885: 4. Type locality: Heng-shan-hsien, Hunan, China. Gredler 1887: 283, pl. 11, figs 1–3.

Moellendorffia (Moellendorffia) hensaniensis: Zilch 1966: 210.

*Moellendorffia hensaniensis*: Zilch 1974: 194. Richardson 1985: 185.

**Current taxonomic status.**

*Moellendorffia*. Valid species.

**Type specimens.**

Lectotype NHMW 15795 (Fig. 5B) and paralectotype SMF 50076/1 (one shell, Fig. 5C) from Hensan, China.

**Remarks.**

The lectotype was designated in Zilch (1974: 194) and illustrated for the first time in this study.

**9. *loxotata* (Mabille, 1887)**

*Helix loxotata*Mabille 1887a: 5. Type locality: Tonkin.

*Proctostoma loxotatum*: Mabille 1887b: 102–104, pl. 1, figs 1–3.

*Moellendorffia loxotata*: Richardson 1985: 185.

*Moellendorffia loxotata loxotata*: Schileyko 2011: 44.

**Current taxonomic status.**

*Moellendorffia*. Valid species.

**Type specimens.**

Syntype MNHN-IM-2000-2071 (one shell, Fig. 5D).

**10. *messageri* (Bavay & Dautzenberg, 1899)**

Helix (Moellendorffia) messageri Bavay & Dautzenberg, 1899: 33–35, pl. 1, fig. 5. Type locality: entre Lang-Son et That-Khé [That Khe Town, Trang Dinh District, Lang Son Province, Vietnam].

*Moellendorffia messageri*: Richardson 1985: 185, 186. Schileyko 2011: 44.

**Current taxonomic status.**

*Moellendorffia*. Valid species.

**Type specimens.**

Syntype MNHN-IM-2000-1939 (one shell, Fig. 5E).

**11. *sculpticoncha* (Zilch, 1951)**

Helix (Polygyra) trisinuata var. sculptilisMöllendorff 1884: 310, 311, pl. 7, fig. 4 [non Bland 1858: 279]. Type locality: Lo-fou-shan, Guang-dung [Guangdong, China].

*Moellendorffia trisinuata sculpticoncha*Zilch 1951: 86 [nomen novum for Helix (Polygyra) trisinuata var. sculptilis Möllendorff, 1884]. Zilch 1966: 211, pl. 6, fig. 53. Richardson 1985: 186.

**Current taxonomic status.**

*Moellendorffia trisinuata sculpticoncha*. Accepted subspecies (Zilch 1966).

**Type specimens.**

Lectotype SMF 8331/1 (Fig. 5F) and paralectotypes SMF 8332/3 (three shells), SMF 27142/4 (four shells) from Lo-fou-shan, Guang-dung, China.

**Remarks.**

The lectotype was designated in Zilch (1966: 211).

**12. *sculptilis* Möllendorff, 1884**

**Remarks.**

see under “*sculpticoncha*”.

**13. *spurca* (Bavay & Dautzenberg, 1899)**

Helix (Moellendorffia) spurcaBavay and Dautzenberg 1899: 31–33, pl. 1, fig. 4. Type locality: environ de Bac-Kau [Bac Quang, Than Uyen District, Lai Chau Province, Vietnam].

*Moellendorffia spurca*: Richardson 1985: 186.

*Moellendorffia spurca spurca*: Schileyko 2011: 44.

**Current taxonomic status.**

*Moellendorffia*. Valid species.

**Type specimens.**

**Figure 6.**
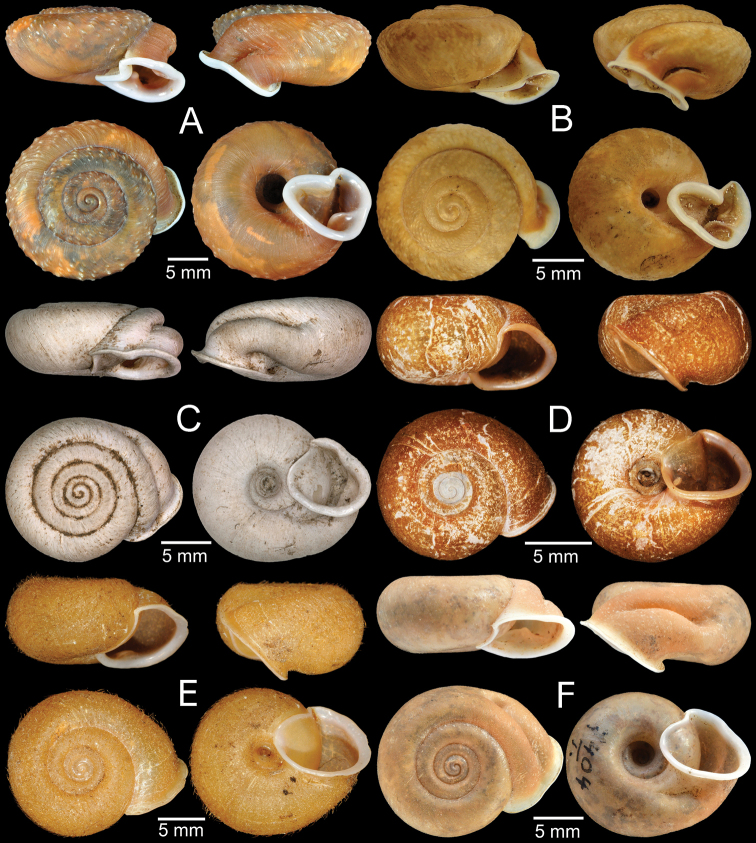
**A***Moellendorffia spurca*, syntype MNHN-IM-2000-1992 **B***Moellendorffia trisinuata*, syntype ZMB 7620 **C***Trichelix biscalpta*, syntype MCZ 167125 **D***Trichelix diminuta*, lectotype ANSP 90049 **E***Trichelix eucharista*, lectotype ANSP 81221 **F***Trichelix hiraseana*, lectotype SMF 7404/1 of *Stegodera helleri*. Photo: AJ Baldinger (**C**).

Syntype MNHN-IM-2000-1992 (one shell, Fig. 6A).

**14. *trisinuata* (Martens, 1867)**

*Helix trisinuata*Martens 1867: 50, 51. Type locality: Hongkong [Hong Kong].

Moellendorffia (Moellendorffia) trisinuata trisinuata: Zilch 1966: 210, 211.

*Moellendorffia trisinuata*: Richardson 1985: 186.

**Current taxonomic status.**

*Moellendorffia*. Valid species.

**Type specimens.**

Syntype ZMB 7620 (one shell, Fig. 6B) from Hongkong.

**II. Genus *Trichelix* Ancey, 1887**

**Type species.**

*Helix horrida* Pfeiffer, 1863; by original designation.

**Diagnosis.**

Shell flattened to concave, spire shrunken and umbilicate. Periostracum covered with short hairs. Last whorl well rounded and descending anteriorly. Aperture subcircular, without barrier or with barriers inside, and externally marked with furrows. Parietal callus thin, with cord at margin or a little elevated to form nodule; two palatal lamellae arranged spirally; one columellar lamella.

**Remarks.**

The genus *Trichelix* s.l. can be distinguished from *Moellendorffiella* by having concave spire, and short to long periostracal hairs, while *Moellendorffiella* have flat spire and without periostracal hair. The Central Ryukyu-*Trichelix* have a thin parietal callus with cord and very weak furrows below the periphery, and the continental-*Trichelix* have an elevated parietal callus with a nodule, and there are one or two furrows (arranged spirally) on the upper periphery. In comparison, *Moellendorffiella* has a thin parietal callus and one furrow on periphery.

**15. *biscalpta* (Heude, 1885)**

*Helix biscalpta*Heude 1885: 113, pl. 29, fig. 10. Type locality: Tchen-k’eou [Chengkou, Chongqing, China].

*Moellendorffia biscalpta*: Richardson 1985: 183.

Moellendorffia (Trichelix) biscalpta: Minato 2011: 25, fig. 3h.

**Current taxonomic status.**

*Trichelix*. Valid species.

**Type specimens.**

Syntype MCZ 167125 (two shells, Fig. 6C).

**Remarks.**

The original description does not clearly state how many specimens were available to the author, and a unique name-bearing type was not explicitly designated. Heude’s (1885) original description included a single illustration and one set of shell measurements. Johnson (1973: 17) used the term “paratypes” for a lot of two shells from the MCZ collection, but this does not constitute a valid holotype designation (ICZN 1999: Articles 73.1.1 and 73.2 and Recommendation 73F). The MCZ museum registration book states “Cotype”; these are also considered to be syntypes.

**16. *diminuta* (Pilsbry & Hirase, 1905)**

*Moellendorffia eucharistus diminuta*Pilsbry and Hirase 1905: 710. Type locality: Koniya, Oshima, Osumi [Koniya-Setouchi, Oshima District, Kagoshima Prefecture, Japan].

*Moellendorffia diminuta*: Baker 1963: 245.

*Moellendorffia eucharistus diminuta*: Richardson 1985: 184.

Moellendorffia (Trichelix) diminuta: Minato 1971: 36–38, figs 2, 10–12. Minato 1980: 190, fig. 2. Minato 2011: 28, fig. 5c–f.

**Current taxonomic status.**

*Trichelix*. Valid species.

**Type specimens.**

Lectotype ANSP 90049 (Fig. 6D) and paralectotypes ANSP 452028 (three shells).

**Remarks.**

The lectotype was designated by Baker (1963: 245).

**17. *eucharista* (Pilsbry, 1901)**

*Chloritis eucharistus*Pilsbry 1901: 347, 348. Type locality: Oshima [Oshima District, Kagoshima Prefecture, Japan].

Moellendorffia (Trichelix) eucharistus: Habe 1957: 8, 9, pl. 1, figs 3–8. Minato 1971: 36, figs 1, 7–9. Minato 1980: 190, fig. 1. Minato 2011: 26, 28, figs 2a, b, 5a, b.

*Moellendorffia eucharista*: Richardson 1985: 184.

*Trichelix eucharistis*: Schileyko 2003: fig. 1950b, c. (incorrect subsequent spelling)

**Current taxonomic status.**

*Trichelix*. Valid species.

**Type specimens.**

Lectotype ANSP 81221 (one shell, Fig. 6E) from Oshima, Osumi.

**Remark.**

The lectotype was designated in Baker (1963: 245). In the original publication, the type locality was recorded as “Oshima” (=Island) which cannot be precisely located. The original label accompanying the lectotype states “Oshima, Osumi” (= historical name of Kagoshima). Habe (1957) examined the radula and genital anatomy based on a specimen from Amami Oshima, Kagoshima. Therefore, the type locality of this species is probably in the area of the Amami Islands, Kagoshima Prefecture.

**18. *helleri* (Rolle, 1911)**

Stegodera (Trichelix) helleriRolle 1911: 31, 32. Type locality: Toyenmongai auf Formosa [Dong-yuan-men-jie, Tainan City, Taiwan]. Zilch 1966: 211, pl. 6, fig. 57.

*Moellendorffia hiraseana helleri*: Richardson 1985: 185.

**Current taxonomic status.**

*Trichelix*. Synonym of *Trichelix hiraseana* (see Zilch 1966).

**Type specimens.**

Lectotype SMF 7404/1 (Fig. 6F) and paralectotypes SMF 156134/4 (four shells) from Toyenmongai, Formosa. Possible paralectotype NHMUK 20040594 (four shells).

**Remarks.**

Zilch (1966) assumed the SMF 7404 ex. H. Rolle as the holotype. However, there was no unique name-bearing type fixed in the original publication. Hwang (2014: 25) subsequently designated SMF 7404 as the lectotype.

**19. *hiraseana* Pilsbry, 1905**

Moellendorffia (Trichelix) hiraseanaPilsbry 1905: 66, 67, pl. 2, figs 4–6. Type locality: Hotawa, Taiwan. Zilch 1966: 211. Minato 2011: 25, figs 3g, 5h.

*Moellendorffia hiraseana*: Richardson 1985: 185.

**Current taxonomic status.**

*Trichelix*. Valid species.

**Type specimens.**

**Figure 7.**
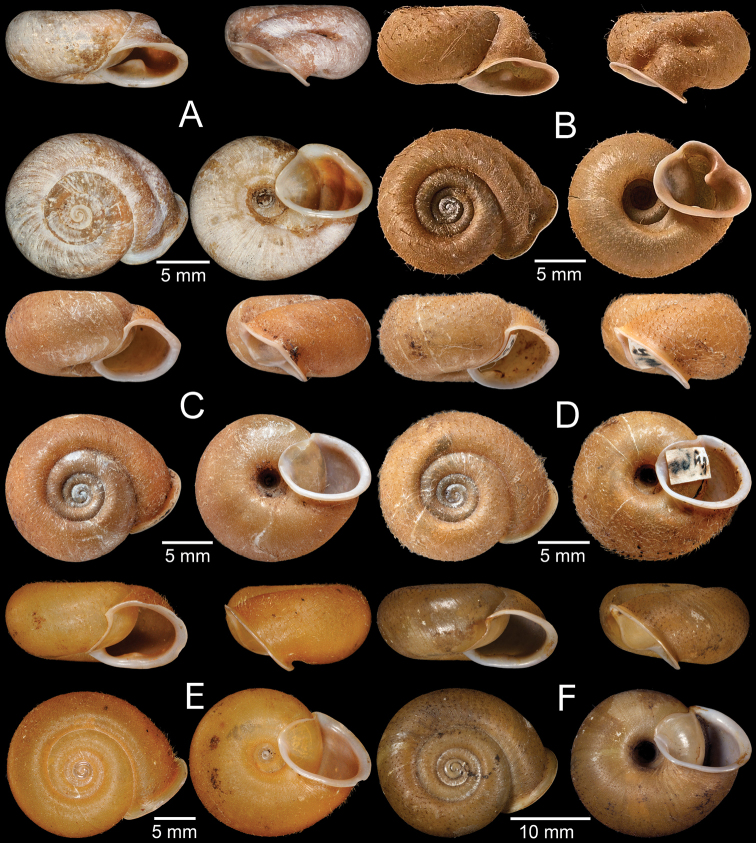
**A***Trichelix hiraseana*, lectotype ANSP 89999 **B***Trichelix horrida*, lectotype NHMUK 20200202/1 **C***Trichelix eucharista*, syntype NHMUK 19991540 of *Chloritis malangensis***D***Trichelix oshimana*, syntype NHMUK 1922.8.29.83 **E, F***Trichelix tokunoensis***E** lectotype ANSP 87680 and **F** paralectotype ANSP 90048.

Lectotype ANSP 89999 (Fig. 7A).

**Remarks.**

Pilsbry (1905) clearly stated that there were two specimens in his lot. The lectotype was designated in Baker (1963: 245).

**20. *horrida* (Pfeiffer, 1863)**

*Helix horrida*Pfeiffer 1863[“1862”]: 272, pl. 36, fig. 15. Type locality: Lao Mountain, Camboja [Cambodia or Laos].

*Moellendorffia horrida*: Richardson 1985: 285. Inkhavilay et al. 2019: 105, figs 53f, 54a, 58h.

**Current taxonomic status.**

*Trichelix*. Valid species.

**Type specimens.**

Lectotype NHMUK 20200202/1 ex. Cuming coll. (Fig. 7B), present designation, and paralectotypes NHMUK 20200202/2 to 20200202/3 ex. Cuming coll. (two shells).

**21. *malangensis* (Bullen, 1905)**

*Chloritis malangensis*Bullen 1905: 192, pl. 11, fig. 2. Type locality: Malang, Java [error]. Gude 1907: 228.

*Moellendorffia eucharista malangensis*: Richardson 1985: 184.

**Current taxonomic status.**

*Trichelix*. Synonym of *Trichelix eucharista* (see Gude 1907: 228).

**Type specimens.**

Syntypes NHMUK 19991540 (two shells, Fig. 7C).

**Remarks.**

Ancey (1906: 128) stated that specimens sent by Mr Rouyer were often with doubtful or inaccurate locality records, where *C. malangensis* Bullen, 1905 was described based on Mr Rouyer’s collection. The type locality was mentioned as “Malang Java,” which is erroneous and should be ignored (Ancey 1906; Gude 1907). Ancey (1906: 128) also noticed this species was similar to *Moellendorffia eucharista* (Pilsbry, 1901) and does not occur in Java. Gude (1907: 228) compared the type specimen of *C. malangensis* with the *Moellendorffia eucharista* (Pilsbry, 1901) from Japan and found no differences in any of the shell characters.

**22. *oshimana* (Gude, 1901)**

*Chloritis oshimana*Gude 1901: 157, 158, figs 1–4. Type locality: Oshima, Loo-Choo Isles [Amami Islands, Kagoshima Prefecture]. Minato 2011: 26.

*Moellendorffia eucharista oshimana*: Richardson 1985: 184.

**Current taxonomic status.**

*Trichelix*. Synonym of *Trichelix eucharista* (see Minato 2011: 26).

**Type specimens.**

Syntype NHMUK 1922.8.29.83 (one shell, Fig. 7D).

**Remark.**

Gude (1901: 158) noted that the collection locality was from Oshima, Osumi Province. The type locality of this species is probably in the area of the Amami Islands of Kagoshima.

**23. *tokunoensis* (Pilsbry & Hirase, 1905)**

*Moellendorffia eucharistus tokunoensis*Pilsbry and Hirase 1905: 710. Type locality: Tokunoshima, Osumi [Tokunoshima Island, Oshima District, Kagoshima Prefecture, Japan].

*Moellendorffia tokunoensis*: Baker 1963: 247.

*Moellendorffia eucharista tokunoensis*: Richardson 1985:184, 185.

Moellendorffia (Trichelix) tokunoensis: Minato 1971: 38, 39, figs 3, 4–6. Minato 1980: 190–192, fig. 3. Minato 2011: 28, 29, fig. 5g.

**Current taxonomic status.**

*Trichelix*. Valid species.

**Type specimens.**

Lectotype ANSP 87680 (Fig. 7E) and paralectotypes ANSP 90048 (two shells, Fig. 7F), ANSP 460394 (one shell) from Tokunoshima, Osumi.

**Remarks.**

The original description did not clearly state how many specimens were available to Pilsbry, although he stated “Types No. 90,048, A. N. S. Phila., from No. 1,207 of Mr. Hirase’s collection.” Later, Baker (1963: 247) designated the ANSP 87680 ex. Hirase no. 1207 lot as the lectotype. This designation is still valid unless there is evidence that ANSP 87680 lot did not form part of the type series (ICZN 1999: Articles 72.1 and 74.2).

**III. Genus *Moellendorffiella* Pilsbry, 1905**

Moellendorffia (Moellendorffiella)Pilsbry 1905: 65. Zilch 1960: 612. Zilch 1966: 211.

*Moellendorffiella*: Schileyko 2003: 1513.

**Type species.**

Helix (Moellendorffia) erdmanni Schmacker & Boettger, 1894; monotypy.

**Diagnosis.**

Shell flattened and umbilicate. Periostracum thin, corneous. Last whorl shouldered and descending anteriorly. Aperture subcircular with barriers inside and externally marked with furrows. Parietal callus thin; one palatal lamella; one columellar lamella.

**Remarks.**

The genus *Moellendorffiella* differs from *Moellendorffia* in having one furrow on periphery, parietal callus thin, and without periostracal hair. While, *Moellendorffia* has one or two furrows on periphery, parietal callus elevated with nodule and short to long periostracal hairs.

**24. *erdmanni* (Schmacker & Boettger, 1894)**

Helix (Moellendorffia) erdmanniSchmacker and Boettger 1894: 173, 174, pl. 9, fig. 8. Type locality: China.

Moellendorffia (Moellendorffiella) erdmanni: Zilch 1966: 211, pl. 6, fig. 55.

*Moellendorffia erdmanni*: Richardson 1985: 184.

*Moellendorffiella erdmanni*: Schileyko 2003: 1513, fig. 1951.

**Current taxonomic status.**

*Moellendorffiella*. Valid species.

**Type specimens.**

**Figure 8.**
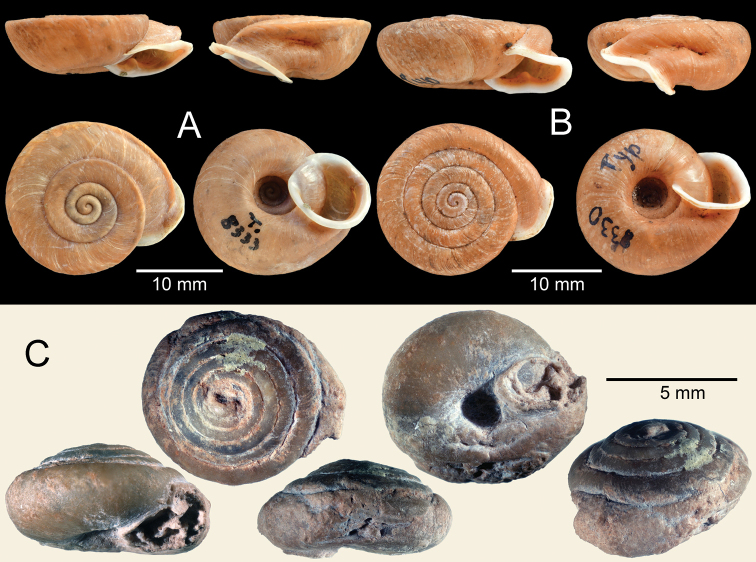
**A***Moellendorffiella erdmanni*, lectotype SMF 8333/1 **B***Moellendorffiella faberiana*, holotype SMF 8330/1 **C**Moellendorffia ? polygyrella, holotype NIGPAS 36428. Photo: T Yü (**C**).

Lectotype SMF 8333/1 (Fig. 8A) and paralectotype SMF 8334 (one shell) from Chang-yang, Hupei, China.

**Remarks.**

The lectotype was designated in Zilch (1966: 211).

**25. *faberiana* (Möllendorff, 1888) comb. nov.**

*Helix faberiana*Möllendorff 1888: 39, 40. Type locality: Omi, Sytshuan, 1000 m. alt. [in the area of Sichuan, China].

Moellendorffia (Trichelix) faberiana: Zilch 1966: 211, pl. 6, fig. 56.

*Moellendorffia faberiana*: Richardson 1985: 185.

**Current taxonomic status.**

*Moellendorffiella*. Valid species.

**Type specimens.**

Holotype SMF 8330/1 (Fig. 8B) and paratype SMF unnumbered (one juvenile in the same holotype lot) from Berg Omi, Szechwan, China.

**Remarks.**

The distinguishing characters are depressed conic spire, aperture with elevated parietal callus, furrows on periphery and below periphery. Therefore, we move this species to the genus *Moellendorffiella*.

### Species inquirenda

***mariae* (Nobre, 1909)**

Stegodera (Moellendorffia) mariaeNobre 1909: 79. Type locality: Lucira, dist. de Benguella [Lucira Communes, Namibe Province, Angola].

*Moellendorffia mariae*: Richardson 1985: 185.

**Current taxonomic status.**

Not a member of *Moellendorffia*, *Trichelix*, or *Moellendorffiella*.

**Type specimens.**

The type specimen could not be located.

**Remarks.**

This nominal species was described by Nobre (1909) based on material collected from Angola on the west coast of Africa. Based on the shell morphology, Nobre (1909) attributed this taxon to the Southeast Asian endemic genus Stegodera (Moellendorffia). Later, Richardson (1985) placed this species under the genus *Moellendorffia*. However, the record of *Moellendorffia* on the east coast of Africa (Ethiopian Realm) are far outside of the known range of the genus. Thus, further study and anatomical examination are needed to relocate this nominal species into the suitable nominal genus, very probably a *Sculptaria* Pfeiffer, 1855 (Sculptariidae).

***polygyrella* Yü, 1982**

Moellendorffia ? polygyrella Yü in Yü et al. 1982: 19, 20, pl. 5, figs 10–14. Type locality: Late Cretaceous and Early Tertiary red series of Xuaneheng, Langxi and Nanling, Southern Anhui.

**Current taxonomic status.**

*Moellendorffia*. Valid species.

**Type specimens.**

Holotype NIGPAS 36428 (one shell: Fig. 8C) from Xuancheng, Luojing, Anhui, China.

**Remarks.**

The species was described based on one specimen. The holotype has a relatively small shell (shell width 8 mm) compared to other recent congeners. This species possesses a smooth shell surface and a narrow umbilicus. The outer surface of the last whorl probably has one spiral furrow on the periphery and two spiral furrows below the periphery. These characters suggest the possibility that it is closely related to the genus *Traumatophora* Ancey, 1887 (see Wu 2019 for a comparison).

## Supplementary Material

XML Treatment for Trichelix

XML Treatment for Trichelix horrida
